# Throat Ail

**Published:** 1877-06

**Authors:** 


### THROAT AIL.

The first most frequent cause of this malady, in our opinion, is indigestion, the lining of the throat being a continuation of the lining of the stomach—one source of nerve-supply to both throat and stomach, being also identical.

There is reason to believe that the second most frequent origin of this troublesome, mischievous and dangerous ailment, is the irregular and injudicious exercise of the vocal organs on the part of singers, clergymen, and other speakers.

The muscles of the human body are under the same general physical laws; they are all developed and strengthened by moderate, regular exercise. If a man runs a hundred yards to-day it will tire him—if he do it a week hence the same result will be observed, for successive months. But if he runs a hundred yards to-day, and repeats it day after day, we all know that, if in ordinary health, the ease with which that race can be performed will become greater and greater, until it can be done without a scarcely appreciable effort. And so it would assuredly be if clergymen would speak in public, in moderation, every day, and when this is not practicable, I have advised the daily reading out aloud in a conversational tone, with the same animation usual to the delivery of a sermon.

The non-use or the spasmodic exercise of any muscle in the human body, will result injuriously, if persevered in.

Another item is of important consideration, and it is a point, too, on which every public speaker should keep his eye steadily fixed. It is not the irregular exercise of the vocal organs, of itself, which originates most of the difficulty; it is that such exercise leaves the throat in a heated and debilitated condition, and in that condition, being suddenly exposed to an altered temperature, injury is the result, on principles precisely the same as are in operation under circumstances of checked perspiration. Single injuries of such a nature would be comparatively harmless, but when repeated weekly for successive years, it is not strange that troublesome symptoms are a permanent result. That clergymen more often suffer from Throat Ail than other public speakers, arises in part from the fact that the exercises are more artificial than those of the lawyer, the legislator or the congressman, but there is a circumstance which we consider a more decided element in producing these morbid phenomena, although we do not at present remember to have seen it alluded to by any medical writer at home or abroad, but which we think will at once strike the reflecting reader as being quite sufficient. The clergyman changes the circumstances under which his public services are performed sooner than any other class of public men. Within ten or fifteen minutes after his discourse is delivered in a close and heated and vitiated apartment, and while yet the whole system, mind and body, is in a state of comparative debility and exhaustion from the two hours' previous effort, he is in a wholly different atmosphere, in the street or on the road, breathing an air, oftentimes cold and damp, raw and chilly, dashing it in at each inhalation, through an open mouth, on a surface of throat equal to a foot square. The lawyer and the legislator usually remain for a considerable time after their efforts, thus allowing the parts to cool and to recover their tone and elasticity. Some of the most intractable and fatal forms of throat disease which have ever fallen under our notice, have arisen from riding on horseback or open vehicle, or walking on foot against a cold wind immediately after preaching. The remedy suggests itself to every thoughtful reader—

1st. Do not expose yourself to an external atmosphere after speaking, until the system is cooled down.

2nd. Or if you must go out soon after, bundle up well, keep your mouth resolutely closed, so as to heat the air before it gets to the throat, by sending it along the circuit of the nose, and walk rapidly, so as to keep up a vigorous circulation.

